# Determination of sediment sources following a major wildfire and evaluation of the use of color properties and polycyclic aromatic hydrocarbons (PAHs) as tracers

**DOI:** 10.1007/s11368-023-03565-0

**Published:** 2023-07-04

**Authors:** K. A. Kieta, P. N. Owens, E. L. Petticrew

**Affiliations:** 1 Natural Resources and Environmental Studies Program, University of Northern British Columbia, Prince George, BC Canada; 2 Department of Geography, Earth and Environmental Sciences, University of Northern British Columbia, Prince George, BC Canada

**Keywords:** Sediment fingerprinting, Conservative behavior, Nechako River, Road-deposited sediment, Wildfire

## Abstract

**Purpose:**

This research aimed to determine if a severe wildfire caused changes in the source of sediment being delivered to downstream aquatic systems and evaluate the use of polycyclic aromatic hydrocarbons (PAHs) and color properties as tracers.

**Methods:**

Sediment samples were collected from 2018 to 2021 in three tributaries impacted by the 2018 Shovel Lake wildfire and from two sites on the mainstem of the Nechako River, British Columbia. Source samples were collected from burned and unburned soils as well as from channel banks and road-deposited sediment. Samples were analyzed for color properties and for the 16 US Environmental Protection Agency priority PAHs. After statistical tests to determine the conservatism and ability to discriminate between sources by the tracers, the MixSIAR unmixing model was used, and its outputs were tested using virtual mixtures.

**Result:**

In the tributaries, burned topsoil was an important contributor to sediment (up to 50%). The mainstem Nechako River was not influenced as significantly by the fires as the greatest contributor was banks (up to 89%). The color properties provided more realistic results than those based on PAHs.

**Conclusion:**

In smaller watersheds, the wildfire had a noticeable impact on sediment sources, though the impacts of the fire seemed to be diluted in the distal mainstem Nechako River. Color tracers behaved conservatively and discriminated between contrasting sources. Due to their low cost and reliability, they should be considered more widely. While PAHs did not work in this study, there are reasons to believe they could be a useful tracer, but more needs to be understood about their behavior and degradation over time.

**Supplementary Information:**

The online version contains supplementary material available at 10.1007/s11368-023-03565-0.

## Introduction

Wildfires are an important natural disturbance in many ecosystems because they rejuvenate forests, but in parts of the world, including North America, the size and severity of fires have begun to increase significantly (Kasischke and Turetsky 2006; Dennison et al. 2014; Abatzoglou and Williams 2016; Iglesias et al. 2022). In the province of British Columbia, Canada, fires in 2017 and 2018 burnt over 1 million hectares (i.e., > 10,000 km^2^), while the 2021 season saw over 800,000 hectares burn, which represent the three largest wildfire years on record (Province of BC 2022). Many of these wildfires, often referred to as megafires, have dramatic impacts on soils (González-Pérez et al. 2004; Certini 2005; Mataix-Solera et al. 2011) and on water quantity and quality (Shakesby and Doerr 2006; Mahat et al. 2016; Leonard et al. 2017; Hohner et al. 2019). In addition to the retardants used for fire suppression that are deposited onto soils, fires themselves can produce toxic compounds (Abdel-Shafy and Mansour 2016; Maletić et al. 2019) and nutrient-laden ash and soils (Baird et al. 1999; Bodí et al. 2014). Additionally, there is often an increase in erosion post-fire due to the elimination of surface vegetation and because of the frequent occurrence of a hydrophobic layer of soil that forms after the condensation of gasses created by burning vegetation onto soil particles (Doerr et al. 1996, 2000; Huffman et al. 2001). This increase in erosion is one of the greatest water quality concerns in wildfire-impacted watersheds, and can include both rill and sheet erosion, as well as more catastrophic landslides and mudslides (Di Napoli et al. 2020; Tillery and Rengers 2020). The increase in sediment mobilization and transport in burned watersheds can be detrimental not only because sediment can have a harmful effect on the aquatic habitat, but also because sediment is a vector for nutrients (e.g., N and P) and contaminants (e.g., metals, fire retardants) (Bladon et al. 2008; Abdel-Shafy and Mansour 2016; Campo et al. 2017). Furthermore, increased fine sediment into receiving watercourses following wildfire can be problematic from the context of water resources, such as drinking water supplies and associated water treatment (Emelko et al. 2011; Smith et al. 2011b).

However, the erosional and water quality responses are not the same in every area impacted by fire as it is heavily dependent on the (i) antecedent soil conditions, (ii) local soil type and vegetation composition, (iii) magnitude and frequency of precipitation post-fire, and (iv) wildfire severity (Neary et al. 2005; Rust et al. 2019). Additionally, there is not a direct correlation between upland erosion and downstream changes in water quality due to the differences in connectivity from the terrestrial to the aquatic ecosystem, in part due to variations in topography (Fryirs 2013; Vercruysse et al. 2017; Wohl et al. 2019). For these reasons, it can be difficult to predict the degree to which different parts of a burnt watershed will contribute sediment and associated chemicals to the river channel network, and how this will change over time as the soil and vegetation recover.

The sediment source fingerprinting approach is widely used across varying landscapes to determine the primary sources of sediment in watersheds of different sizes, topography, and land cover/land use (see reviews by Walling 2013; Owens et al. 2016; Collins et al. 2017, 2020). A myriad of tracers has been used including fallout radionuclides such as cesium-137 (^137^Cs) and lead-210 (^210^Pb), geochemical elements, and a range of physical tracers such as color and mineral magnetism (Haddadchi et al. 2013). As the field has grown, researchers have continued to search for new tracers for a variety of reasons. Some tracers have been investigated because of their potential ability to differentiate between closely linked sources, such as the use of compound-specific stable isotopes (CSSIs) for differentiating between specific crop types (e.g., Reiffarth et al. 2019), or because of emerging technology, such as eDNA (e.g., Evrard et al. 2019b). However, while many of these tracers have successfully been used for a long time, including following wildfires (for a review see Smith et al. 2013), there is also a need for additional tracers that can be utilized in wildfire-impacted watersheds specifically.

Color is a simple and inexpensive property to measure in soil and sediments and thus has been used with increasing frequency in fingerprinting studies (e.g., Barthod et al. 2015; Pulley and Collins 2021), though very few studies have evaluated color following wildfire (García-Comendador et al. 2020). Polycyclic aromatic hydrocarbons (PAHs) are produced during the combustion of organic material such as trees or other vegetation, but also through the presence and combustion of fossil fuels. They can be mutagenic or toxic to aquatic organisms and some are regulated in surface and drinking water (WHO 2008; CCME 2010). Therefore, PAHs are important to study post-wildfire due to their possible detrimental impacts on aquatic and terrestrial ecosystems (Incardona 2017; Ainerua et al. 2020; Wallace et al. 2020; Kieta et al. 2023a). Furthermore, as PAHs are usually enriched in the surface layers of soils following wildfire (Kim et al. 2003; Choi 2014), they could be a potential tracer for wildfire-specific studies.

In this study, we aimed to determine the suitability of PAHs as a new suite of tracers. We used color as a reference tracer, given its widespread use in fingerprinting studies and recent research showing its stability over time (García-Comendador et al. 2023), while acknowledging that color has only been used once in a wildfire setting (i.e., García-Comendador et al. 2020). Therefore, following a catastrophic wildfire in British Columbia in 2018, this paper aims to (i) determine if burned areas are a major source of fine-grained sediment to the downstream river system and (ii) investigate the utility of PAHs and color properties as sediment tracers in wildfire-impacted watersheds.

## Methods

### Study area

The Nechako River Basin (NRB), located in north-central British Columbia, is a large basin (47,200 km^2^) that is regulated by the Kenney Dam and Skins Lake Spillway, which were built in the 1950s. Approximately 50% of the water that would have made its way through the Nechako River via the Nechako Reservoir is diverted 16 km west, through a tunnel in the Coast Mountains to provide power for an aluminum smelter in Kitimat, on the coast of British Columbia. Flow at the spillway is regulated by Rio Tinto with the primary objective of producing energy. However, in 1987 the Summer Temperature Monitoring Program (STMP) was implemented, which mandates that Rio Tinto maintain water temperatures below 20 °C between approximately July 15 and August 15 each year for the health of migrating fish populations. The NRB supports Sockeye (*Oncorhynchus nerka*) and Chinook salmon (*Oncorhynchus tshawytscha*) as well as the Nechako white sturgeon (*Acipenser transmontanus*), whose recruitment failure has led to their classification as endangered and to the construction of a sturgeon hatchery to bolster their populations (COSEWIC 2003; McAdam et al. 2005).

The region is sparsely populated, with the largest community being Vanderhoof (pop. ~ 4500), but has been heavily impacted by forest harvesting and agriculture. Harvesting was accelerated across the province, and in this region in particular, during the Mountain Pine Beetle (*Dendroctonus ponderosae*) epidemic, which started in the 1990s and affected over 18 million hectares of forest. As a result, the annual allowable cut of trees was doubled in order to salvage the timber (BC Ministry of Forests and Range 2007). The Mountain Pine Beetle epidemic also left a large amount of dead wood on the landscape, which has been a factor in the increase in wildfires in the region in the last decade. The NRB experienced its worst fire season in over a decade in 2018, and one of the largest fires within the basin was the Shovel Lake wildfire, which burned 92,000 hectares (920 km^2^) between July 27 and September 5.

**Fig. 1:**
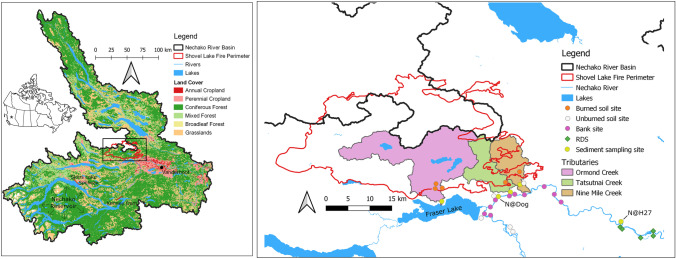
Map of the Nechako River Basin (left) and the Shovel Lake wildfire perimeter (right) with studied tributaries and sampling sites. Note the black box on the NRB watershed map denotes the study area

Three major tributaries to the Nechako River were heavily impacted by the wildfire: Ormond (248 km^2^), Tatsutnai (68.2 km^2^), and Nine Mile Creeks (66.1 km^2^) (Table 1). As shown in Fig. 1, Ormond Creek, the largest catchment, flows directly into Fraser Lake, a large lake (54.3 km^2^) that empties into the Nechako River via the 800-m-long Nautley River. Both Tatsutnai and Nine Mile Creeks empty directly into the Nechako mainstem downstream of the confluence with the Nautley. Table 1 provides more detailed characteristics including drainage area, estimated mean annual discharge, elevation range (Chapman et al. 2018), land cover (GeoBC 2001), and percent burned (BC Forest Analysis and Inventory Branch 2020) for each of the tributaries.

**Table 1:** Major characteristics of the studied tributaries

**Tributary**	**Drainage area (km**^**2**^**)**	**Mean annual discharge (m**^**3**^ **s**^**−1**^)*	**Max/Min elevation (m)**	**Non-forested (%) (%Agri-culture)**^**a**^	**Water and wetlands (%)**	**Forested (%)**^**b**^	**Paved and gravel road length (km)**^**c**^	**%burned (%high, %med, %low, %unburned)**
Ormond	248	1.13	1366/705	2.9 (0)	6.3	90.7	170	93 (29, 58, 10, 3)
Tatsutnai	68.2	0.423	1382/701	19.2 (2.2)	1.6	79.2	10	76 (31, 57, 10, 2)
Nine Mile	66.1	0.421	1286/682	28.9 (2.4)	2.9	68.2	30	47 (17, 44, 16, 22)

*Estimated by Chapman et al. (2018)

^a^Includes agriculture, herbland, shrubland, and exposed land

^b^Includes mixed, broadleaf, and coniferous forests

^c^Includes only known, maintained roads (GeoBC 2017)

The mean annual air temperature in the NRB is 3.7 °C, and the mean annual precipitation is ~ 600 mm, about 200 mm of which falls as snow (Albers et al. 2016). There is a Water Survey of Canada hydrometric station in Vanderhoof, and the mean annual discharge is 114.4 m^3^ s^−1^ with peak discharge occurring during the annual freshet between late May and late June, typically between 350 and 400 m^3^ s^−1^. The parent material in the NRB is primarily gray basalt or sandy till, and the primary soils are brunisols (Cambisol reference soil group, Smith et al. 2011a) beneath a thin surficial humus layer (Cook and Dunn 2006).

### Source and sediment sampling

Soil sampling was undertaken post-fire, when conditions were safe in October 2018, and again in September 2020. In 2018, 10 sites were selected, five burned and five unburned. At the burned sites, the burned organic material (OM) was collected by scraping the soil surface, and this sample consisted mainly of ash as large debris such as burnt pinecones were removed. After collecting the burned OM sample, the burned soil layer (0–2 cm) and the subsurface soil (2–10 cm) were sampled. At the unburned sites, the topsoil (0–2 cm) and subsurface soil (2–10 cm) were collected. In 2020, only the burned sites were re-sampled, and the collection was limited to the topsoil. Multiple (~ 10–15) sub-samples were collected within a 10 m radius at each site and composited in order to ensure enough material was collected for analysis and that a spatially representative sample was collected, while ensuring fire severity and soil type remained consistent. Samples of the burned OM were collected using a hand trowel, while topsoil and subsurface soil samples were collected using a JMC Backsaver soil sampler (JMC, Newton, IA, USA), both of which were cleaned with ethanol and deionized water between sites.

In addition to soils (burned and unburned), the two other main potential sources of sediment to the study rivers are eroding channel banks and, in the case of the mainstem, road-deposited sediment (RDS). RDS is derived from a couple of sources, primarily from the direct deposition of material from vehicles, but also from material that may be left over from winter traction control (e.g., sand and gravels). However, the latter type of material is usually swept in the spring, after the snow has disappeared, while the RDS samples were collected in late summer and fall. The RDS samples do not include material from unpaved or unsealed roads directly. The PAHs associated with RDS are from a variety of sources, including tar-based road sealant, vehicle braking material, and fuel emissions, among others. The RDS samples were collected by sweeping material directly into sample bags using fiber paintbrushes from major road shoulders and also from the road shoulder on two major bridges, all of which were paved. At each of the five RDS sites, five samples were collected and composited, and samples were taken from well-trafficked roads near the suspended sediment sampling sites. Bank samples were collected at locations where the exposed bank was less than 1 m in height and 5 m in length, by using a hand trowel to sample five points at 10 sampling sites, integrating across the height and length of the exposed bank. The 10 sampling sites were collected from an approximately 45 km stretch of the mainstem Nechako River, from the outlet of Fraser Lake to the most downstream site, N@H27. The hand trowels were cleaned with ethanol and deionized water between sites and samples of both banks and RDS were kept in coolers until they were returned to the lab, where they were stored at 4 °C.

Sediment samples were collected from the three tributaries (*n* = 73) and from two sites (*n* = 40) on the mainstem Nechako River, N@Dog and N@H27 (Fig. 1, Sect. 2.1). Sampling occurred from November 2018 to September 2021 during the ice-free period, typically April–October. At each site, two time-integrated passive samplers (for details see Phillips et al. 2000) were attached to cement blocks and placed parallel to the flow on the bed of the river. Care was taken to ensure that sediment resuspended while the samplers were being deployed was washed out of the samplers. Samples were collected bi-weekly from the beginning of the snowmelt (April) until mid-July, corresponding with the start of the STMP. During and after the STMP, samples were collected monthly until the tributaries and main river started to freeze (October/November). Upon retrieval, the samplers were emptied into clean 19-L buckets and transported back to the laboratory at the University of Northern British Columbia (UNBC).

### Laboratory analysis

Sediment samples were left to settle for 48 h, after which the buckets were dewatered, and sediment was placed into clean glassware. Sediment and source samples were sieved to 1 mm to prepare for chemical and physical analysis. While the typical particle size chosen for fingerprinting studies is < 63 μm, for this study, 1 mm was chosen for two reasons. The first is that for PAH analysis, a 10 g dried sample is necessary, which was at times difficult to obtain from sediment samples even when sieving samples to 1 mm. The second is that the NRB has been shown to transport significant quantities of sand (Rood 1998; Northwest Hydraulic Consultants Ltd. 2003) and this size fraction has been identified as one of the stressors for the sturgeon spawning grounds near Vanderhoof (McAdam et al. 2005). Thus, targeting this size class makes sense geomorphologically and ecologically. Samples analyzed for PAHs were sent on ice to AXYS/SGS Laboratories (Sidney, BC). The following 16 US Environmental Protection Agency (EPA) priority PAHs were determined: naphthalene (Nap), acenaphthene (Ace), acenaphthylene (Acy), anthracene (Ant), fluorene (Flu), phenanthrene (Phe), fluoranthene (Flt), pyrene (Pyr), chrysene (Chr), benz[a]anthracene (BaA), benzo[a]pyrene (BaP), benzo[b]fluoranthene (BbF), benzo[j,k]fluoranthenes (BjkF), dibenz[a,h]anthracene (DaA), indeno[1,2,3-cd]pyrene (Ind), benzo[ghi]perylene (BgP). These 16 PAHs were chosen as they are the most common suite of PAHs analyzed in studies concerned with terrestrial and freshwater ecosystems due to the relative ease of analysis, their importance in assessing human and ecosystem health risk, and as a way to narrow down a list of thousands of polycyclic aromatic compounds to a number that is both manageable and comparable across studies (Andersson and Achten 2015). Due to the high cost of PAH analysis (~ $450/sample), only a limited number of sediment samples were analyzed. The subset of sediment samples chosen for analysis was based on season and by existing knowledge of how the stream hydrograph is altered by the dam and spillway. Therefore, the November 2018 sample was analyzed as it was the first opportunity post-wildfire, followed by a focus on the spring snowmelt period in 2019, when it was expected that there would be a first pulse of PAH-laden runoff. In 2020, a sample from the lower elevation melt period (May 8) and higher elevation melt period (July 6) were chosen, as well as a sample from the end of the STMP, when any wildfire-derived sediment that had settled on the Nechako River bottom may have been resuspended with the regulated high flows. Based on the PAH concentrations found during 2019 and 2020, the snowmelt period was the sole focus of the 2021 sample analysis.

Samples were extracted by Soxhlet using dichloromethane after they were fortified with a suite of 17 deuterium-labeled quantification standards. Using a combination of silica and alumina columns, sample extracts were chromatographically cleaned, and then the extracts were reduced in volume and fortified with an internal recovery standard. Samples were measured by gas chromatography with mass spectrometric detection (Agilent 6890N gas chromatography/MSD system). The MS was operated at unit mass resolution in the electron impact ionization mode, and two ions were monitored for all PAH and labeled surrogates. Calibration was verified once every 12 h, and initial calibration was performed on all analytes and labeled surrogate standards using a five-point series. Gas chromatographic separation was attained using a 30 m RTX-5, 30 m, 0.25 mm (i.d.), and 0.25 film thickness GC column. Target PAHs were confirmed when (i) individual chromatographic peak responses were at least three times the background noise level, (ii) were within 3 s of the elution time predicted from the calibration run, (iii) peak centroids from quantification and confirmation ions coincided within 2 s, and (iv) relative ion abundance ratios of the two monitored ions per analyte were within 20% of the corresponding ratio in the calibration run. Their concentrations were calculated using the isotope dilution method and were reported in ng g^−1^. As explained above, due to the high cost and significant mass (10 g dried) needed for PAH analysis, not all of the sediment, source, or bank samples could be analyzed for PAHs. Furthermore, not all PAH samples could also be analyzed for color because PAH analysis is destructive and is undertaken on fresh samples whereas color samples need to be dried for analysis.

Samples analyzed for loss on ignition (LOI), particle size, and color were dried at 40 °C. Particle size analysis was undertaken at UNBC using a Malvern 3000 particle size analyzer (Malvern Panalytical, Malvern, UK) after the samples had been treated with hydrogen peroxide to remove organic matter and sodium hexametaphosphate for dispersion. Loss on ignition was completed by weighing samples before and after ashing them for 4 h at 525 °C.

For color analysis, 7-g samples were placed into 9-mm Petri dishes, sealed with electrical tape, and shipped to the University of Manitoba for analysis. Upon receipt, samples were smoothed, and the visible and near-infrared reflectance was measured by a portable spectroradiometer (ASD FieldSpec Pro, Analytical Spectral Device Inc., CO, USA). For each sample, a white reference was first collected followed by 10 spectra for the source and sediment samples. The average spectra across the 10 scans were calculated, followed by 15 color coefficients (for analytical details, see Barthod et al. 2015). Briefly, spectral reflectance data between 360 and 830 nm across the 10 scans were averaged, and the XYZ color coefficients were calculated (Barthod et al. 2015). The CIE xyY, CIE L*a*b*, CIE L*u*v*, and CIE L*c*h* and RGB color coefficients are derived from the XYZ color system.

### Statistical analysis

Sediment fingerprinting techniques rely on the assumption that the tracers being used act conservatively (i.e., they do not change from source to sink), and that they are able to discriminate between sources (Koiter et al. 2013; Walling 2013). Various methodologies have been proposed to determine the conservative behavior of tracers, and in this study, a three-step procedure was used. First, a simple range test was performed by finding the minimum and maximum for source and sediment samples and removing any tracer where the sediment samples were outside of the range of values for the source samples. Next, the non-parametric Kruskal-Wallis *h*-test was used to determine the discriminatory power of the tracers, and those with a *p* > 0.05 were eliminated. Finally, with the remaining tracers, multiple linear discriminant analyses (LDA) were undertaken to determine if there was an overlap between potential sources. Individual LDAs were run for PAHs and color as well as for the tributaries and for the mainstem due to the inclusion of RDS in the mainstem unmixing model, but not the tributaries. Any individual PAH where more than 10% of samples were below the limit of detection (LOD) was eliminated prior to the range test, and any samples with a concentration below the LOD were replaced by LOD/2 (i.e., the mid-point value between 0 and LOD).

A Bayesian hierarchical mixing model, MixSIAR, was applied to apportion sources of sediment at each site using the tracers identified by the statistical procedures outlined above. MixSIAR is a probabilistic model that uses Markov Chain Monte Carlo (MCMC) sampling within the R workspace (Stock and Semmens 2016). Due to the collection of a single sediment sample at each date, the error structure was “process only,” and “date” was considered a random factor. All models were run with an uninformative prior, a “very long” MCMC run with a chain length of 1,000,000, a burn-in of 500,000, and model convergence was assessed using the Gelman-Rubin diagnostic.

The evaluation of MixSIAR outputs can be undertaken using artificial mixtures, which include those created in the laboratory (e.g., Haddadchi et al. 2014; Gaspar et al. 2019) and those created virtually (e.g., Batista et al. 2022). In this study, virtual mixtures were used due to the ease with which the analysis is undertaken (i.e., mathematically) and due to the high cost of analysis that makes laboratory mixtures prohibitive. Additionally, Batista et al. (2022) found the use of virtual mixtures to be nearly as useful as laboratory mixtures, though in the case of PAHs, an untested tracer, this may not be the case. A full evaluation of the use of virtual mixtures was not the main objective of this paper (see Batista et al. 2022), but to evaluate both model output and subsequently, source discrimination, 15 virtual mixtures for both tributaries and the mainstem sources (*n* = 60) were analyzed for both color and PAHs. Mixtures were created by multiplying the proportion of each source in the mixture by the mean source tracer value. The aim of this study was to determine the primary sources of sediment post-wildfire, and as there is no single metric for evaluating the results from the virtual mixtures, we calculated the percent that the model correctly identified the primary source of sediment at the median, within the CI_50_ and CI_95_, and the absolute error (i.e., predicted value - observed value).

## Results

To assess the potential for differences in particle size between the source and sediment samples, Kruskal-Wallis tests were utilized. This showed that there were no significant differences in the mean particle size (D_50_) between sources and sediments (Table 2), with the exception of Site 44, an unburned soil sampling site. Due to the importance of consistent particle sizes across sediments and sources, and to avoid the use of a particle size correction factor that adds uncertainty to the model (Laceby et al. 2017; Koiter et al. 2018), samples from Site 44 were removed from the analysis. While this may reflect a naturally occurring difference at this site, based on soil mapping (Agriculture and Agri-Food Canada 1974), it is a coarser soil type than much of the burned area and it is similar to other unburned sites and thus, its inclusion would increase uncertainty in the model. There were no significant differences between PAH concentrations nor color coefficients in the subsurface soil samples between burned and unburned sites (i.e., the wildfire did not change these properties), and therefore, they were combined as a single source (unburned soil). Table S1 provides a synthesis of source and sediment samples collected and identifies which samples were used for each MixSIAR analysis.

**Table 2:** Particle size analysis results for source and sediment samples

Sample type	D_10_ (µm)	D_50_ (µm)	D_90_ (µm)	SSA (m^2^ kg^−1^)
Source	2.67 (1.53)	20.5 (11.6)	125.6 (77.7)	429.4 (205.5)
Sediment	2.11 (0.78)	26.4 (19.7)	137.0 (97.5)	436.6 (118.9)

Values reported are mean values (standard deviation). SSA denotes specific surface area

### Determination of sediment sources using color properties

**Fig. 2:**
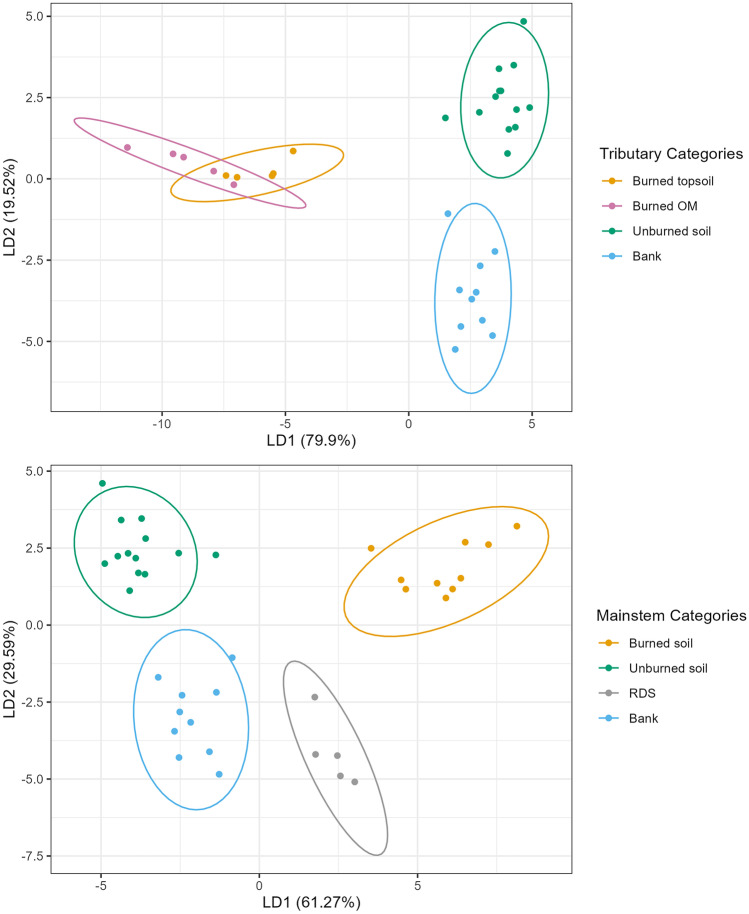
Results from the linear discriminant analysis for tributary (top) and mainstem (bottom) source samples using color parameters

The mean values for source and sediment samples were determined prior to the use of the range and Kruskal-Wallis tests (Table 3). Results from these tests removed six color coefficients, leaving nine coefficients as tracers: X, Y, Z, l, c, b, R, B, G. These variables were included in the LDA with five sources: burned OM, burned topsoil, unburned soil, bank material, and RDS. The results of the LDA showed that there was some overlap between burned OM and burned topsoil in the LDA plot, so for the mainstem sites, they were combined into one source grouping (“burned”), leaving four sources: burned soil, unburned soil, RDS, and bank material (Fig. 2). For the tributary sites, RDS was removed as a source of sediment because there are very few paved roads within the tributaries (Table 1), which is important as road sealants are a common source of PAHs (Baldwin et al. 2020; Van Metre et al. 2022). There was less overlap at the tributary sites between burned OM and burned topsoil, and thus, they were not combined, leaving four sources: burned OM, burned topsoil, unburned soil, and bank material (Fig. 2). A comparison of the color values from the 2018 and 2020 source samples was undertaken, and results showed that there was no significant difference (*p* < 0.05) between years, another line of evidence that color tracers are acting conservatively. Therefore, for all of the MixSIAR analyses using color parameters, the 2018 source sample values were used (Table S1).

**Table 3:** Mean (and standard deviation) for color coefficients, not including values from samples collected in 2020 and 2021, which were not used in MixSIAR analysis

Color coefficient	Sediment	Source
X*	23.0 (5.9)	17.1 (9.0)
Y*	19.7 (5.2)	14.4 (7.6)
Z*	4.9 (1.4)	3.6 (1.8)
x	0.48 (0.005)	0.48 (0.01)
y	0.41 (0.001)	0.41 (0.003)
u	12.0 (1.8)	11.2 (4.7)
v	5.07 (0.75)	4.03 (1.9)
L*	51.0 (6.0)	43.0 (10.8)
a	5.2 (0.99)	5.1 (1.9)
b*	12.5 (1.6)	10.7 (4.5)
h	45.8 (31.4)	37.9 (29.5)
c*	13.6 (1.7)	11.9 (4.9)
R*	133.5 (15.0)	116.5 (30.2)
G*	117.8 (15.5)	98.5 (25.1)
B*	100.0 (13.9)	84.1 (19.5)

*Denotes the coefficients selected for MixSIAR

**Fig. 3:**
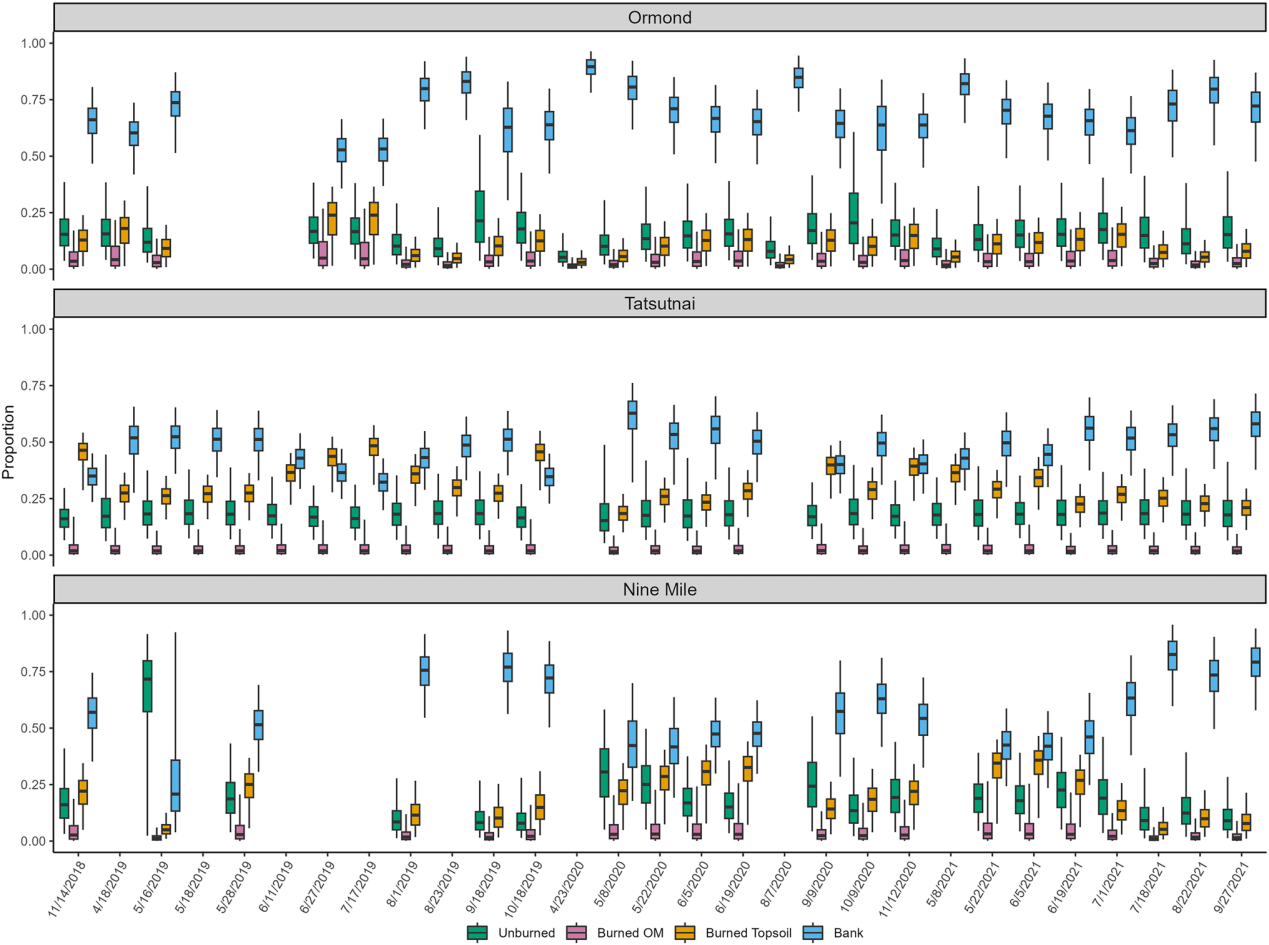
Proportional source contributions to sediment samples collected from tributary sites using color parameters for tracing

Results from Ormond Creek (Fig. 3), the largest watershed of the three tributaries, showed that it had the smallest proportion of sediment derived from burned sources among the tributaries. The greatest proportion of burned topsoil (up to ca. 24%) was derived during the summer of 2019, which coincides with the first period of snowmelt after the wildfire, starting in the valley bottoms (April–May) and then the mountains (June–July). Contributions from burned OM were small, with a maximum of just 5%, in the summer of 2019. The general trend in Ormond Creek was that channel banks were the dominant source, with a maximum contribution of 90% during the spring snowmelt in 2020. Contributions from banks were the highest in the spring (snowmelt period) and fall (heaviest rainfall), with a more mixed signal during the summers. This was particularly true in the first year after the wildfire, but banks became more dominant in the summers by the end of the study period.

Nine Mile and Tatsutnai Creeks are different than Ormond Creek in that they are smaller in size and have similar land use (Table 1). While the results from these watersheds are more similar to each other than to Ormond, there are some noticeable differences. As shown in Fig. 3, in Tatsutnai, banks contributed an overall median of 48% with a range of 32–63%, compared to Nine Mile, where banks contributed an overall median of about 57%, but a range of 21–83%. The starkest difference was in burned topsoil, which ranged at Tatsutnai from 18 to 48% (median = 31%), but at Nine Mile ranged from 5–36% (median = 18%). Contributions from burned OM were noticeably low at both sites, contributing just 1–4%. At Tatsutnai, there was an increase in the contribution from burned topsoil during the late summer months of 2019, which, as noted with Ormond Creek, coincides with the timing of snowmelt in the mountains. Fall 2020 and spring 2021 showed an increase in the proportion derived from burned topsoil after small contributions during the 2020 snowmelt period.

The contribution from unburned soil at Tatsutnai remained consistent throughout the study, ranging from 15 to 18% (Fig. 3). Nine Mile was the least burned watershed (Table 1) and also had the highest range in contribution from unburned soils (8–72%) across the tributaries. Additionally, the contribution from burned topsoil decreased from spring 2019 to fall 2019, which coincided with the heaviest rain of the year. However, this does not necessarily equate to a decreased load of burned topsoil, as the rain could have increased the total load of sediment from all sources to the creek, though load data were not collected in this study to confirm that this occurred. Nine Mile Creek showed a clear trend of a decreasing contribution over time from burned topsoil in the final year of the study (Fig. 3). Also of note is the fewer number of sediment samples collected at Nine Mile Creek (*n* = 20) compared to Ormond (*n* = 26) and Tatsutnai (*n* = 27), which is due to the inability to obtain enough sediment in the passive sampling traps for analysis over the 2-week sampling period.

**Fig. 4:**
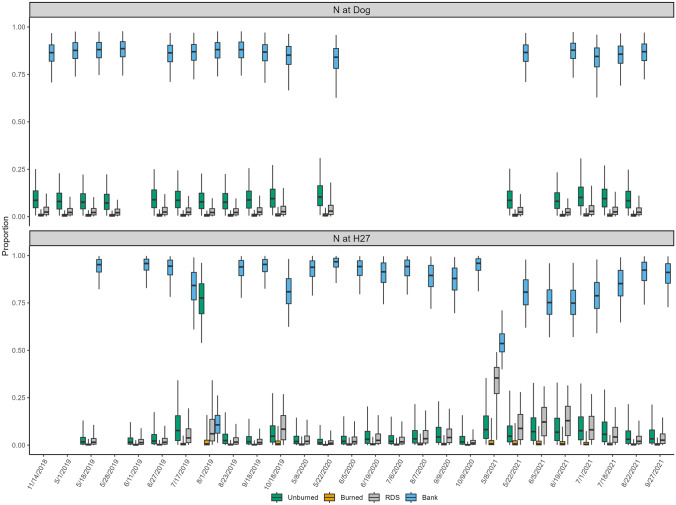
Proportional source contributions to sediment samples collected from the Nechako River mainstem sites using color parameters for tracing

The two mainstem sites, one upstream from the burned tributaries (N@Dog) and one below the burned tributaries (N@H27), were similar in showing very little evidence of contributions from burned material. At both sites, the overall median contribution from burned material was just 1%, with a range of 0–1% (Fig. 4). Unburned soils contributed a median of 9% at N@Dog, slightly higher than the 5% median at N@H27. While the range at N@Dog was just 7–10%, the range at N@H27 was 1–78%, though when the sample from August 2019 is removed, the maximum drops to 8%, as the August sample was the only one where unburned sources were dominant. There was a smaller number of samples collected at N@Dog (*n* = 16), which was in a wider (~ 250 m) section of the river than at N@H27 (*n* = 24), which was narrower (~ 130 m) and has some upstream exposed banks, though they have similar discharge (mean discharge of ~ 114 m^3^ s^−1^ and ~ 117 m^3^ s^−1^ respectively). This discrepancy in sample numbers was particularly clear in 2020, when only one sample could be collected at N@Dog. The overall contribution from RDS was not significantly different between the two mainstem sites, with a median contribution of 3% at N@Dog and a median at N@H27 of 4%, but the major difference was evident in the range of values. At N@Dog, contributions ranged between 2 and 3%, but at N@H27, the site is directly below a bridge on a busy road, and the range was 1–35%, with elevated concentrations particularly pronounced in 2021 (Fig. 4).

### Determination of sediment sources using PAHs as tracers

**Fig. 5:**
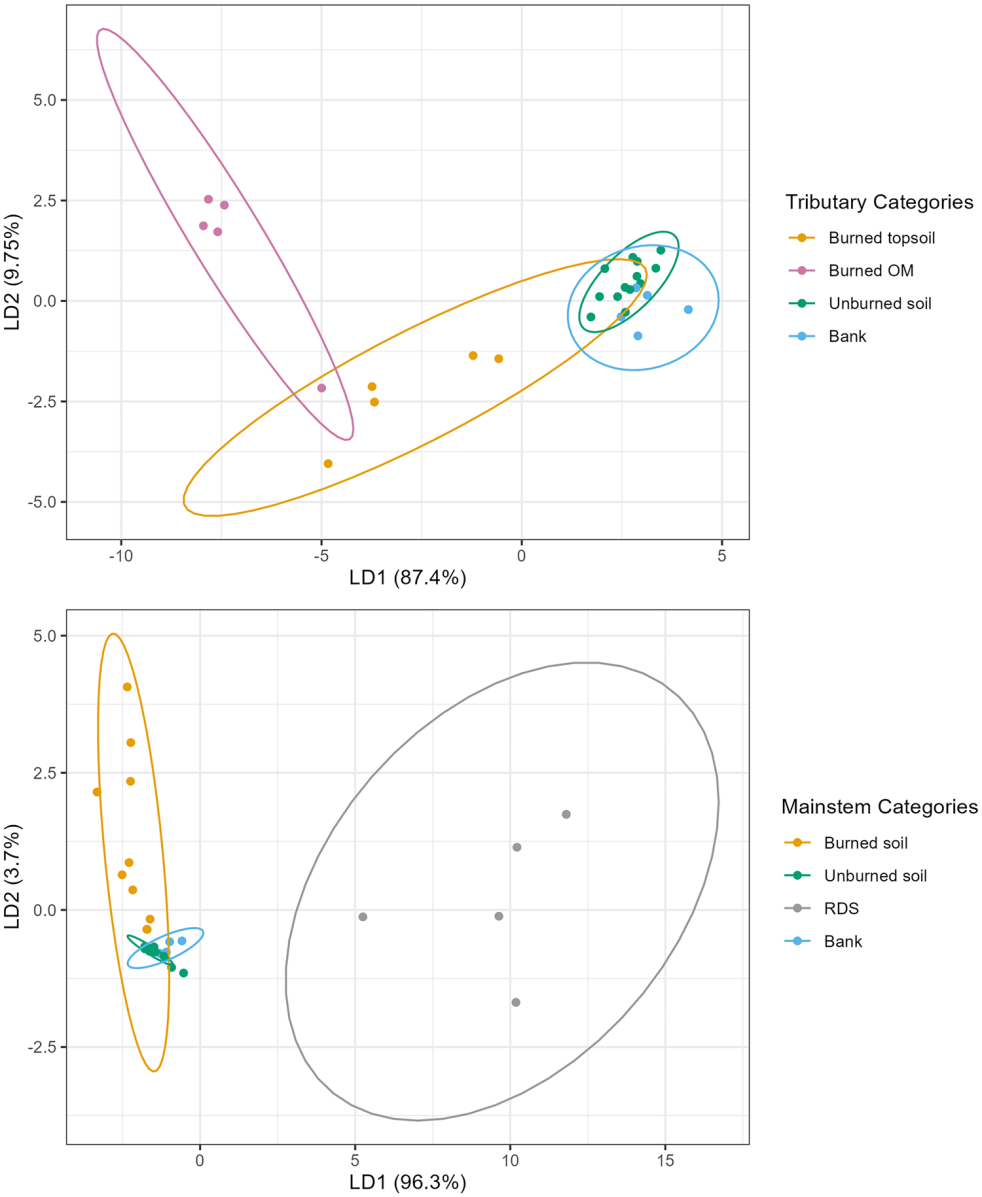
Results from the linear discriminant analysis for tributary (top) and mainstem (bottom) source samples using PAH parameters

Results from the range and Kruskal-Wallis tests removed eight PAH compounds, leaving Ace, Ant, BaA, BghiP, BjkF, Flua, IcdP, and Pyr as potential tracers. With the exception of DaA, which failed the Kruskal Wallis test, the other PAHs were removed because the minimum sediment value was below the minimum source value. The predict function of the LDA was not as accurate with the PAHs as it was for color, as the bank samples had significant overlap with unburned soil and some overlap with the burned topsoil (Fig. 5). However, because these variables passed the range and Kruskal-Wallis tests, and to keep consistency across PAHs and color analysis, they were all included in the MixSIAR analysis. Also, in keeping consistency across the two tracer types, RDS was removed as a source of sediment from the tributary sites, due to the minimal roads within their watersheds (Table S1).

**Fig. 6:**
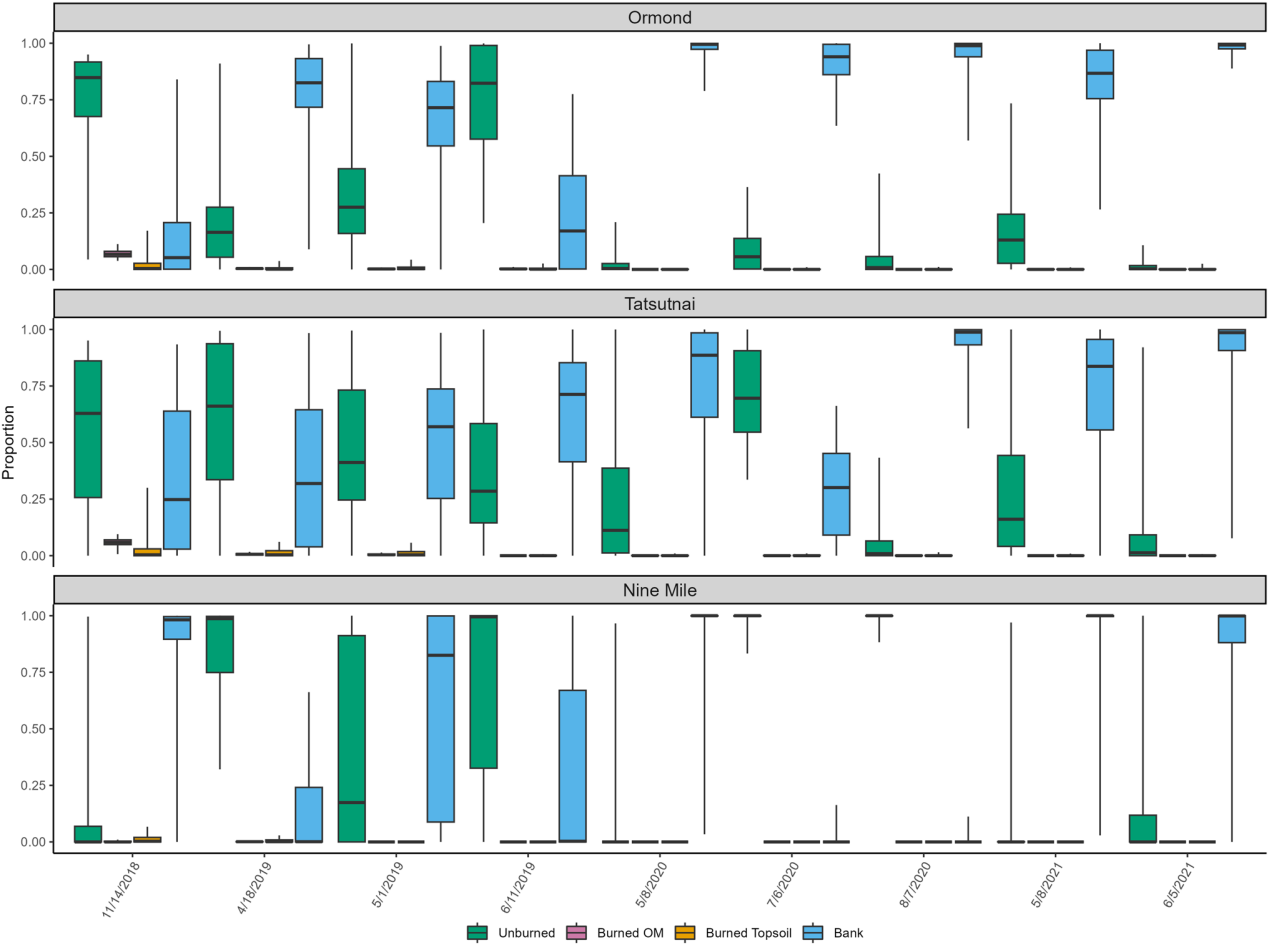
Proportional source contributions to sediment samples collected from tributary sites using polycyclic aromatic hydrocarbon parameters for tracing

The results from MixSIAR using PAHs in the tributaries (Fig. 6) were not consistent with the results using color properties (Fig. 3). At Tatsutnai, the overall median concentration from banks was 71%, ranging from 25 to 99%, and for unburned soil, the overall median was 29%, ranging from 1 to 70%. These two sources made up nearly 100% of the sediment sources throughout the duration of the study, though the banks became dominant over unburned soils by June 2019. There was no meaningful contribution from burned topsoil and burned OM, where the median was < 1% for both sources, with ranges from 0 to 1% and 0 to 6% respectively. At Nine Mile, the results were similar, with unburned soil and banks contributing 98–100% to each sediment sample. Finally, at Ormond Creek, the results showed that either unburned soil or banks were responsible for nearly 100% of the sediment, with banks contributing 100% from May 2020 throughout the rest of the study period. Prior to May 2020, unburned soil contributed 16–82%, with banks making up the remaining contributions. It is worth noting that for all three tributaries, the greatest contribution from wildfire-influenced sources (burned OM and burned topsoil) was for the first sample collected after the wildfire in November 2018.

**Fig. 7:**
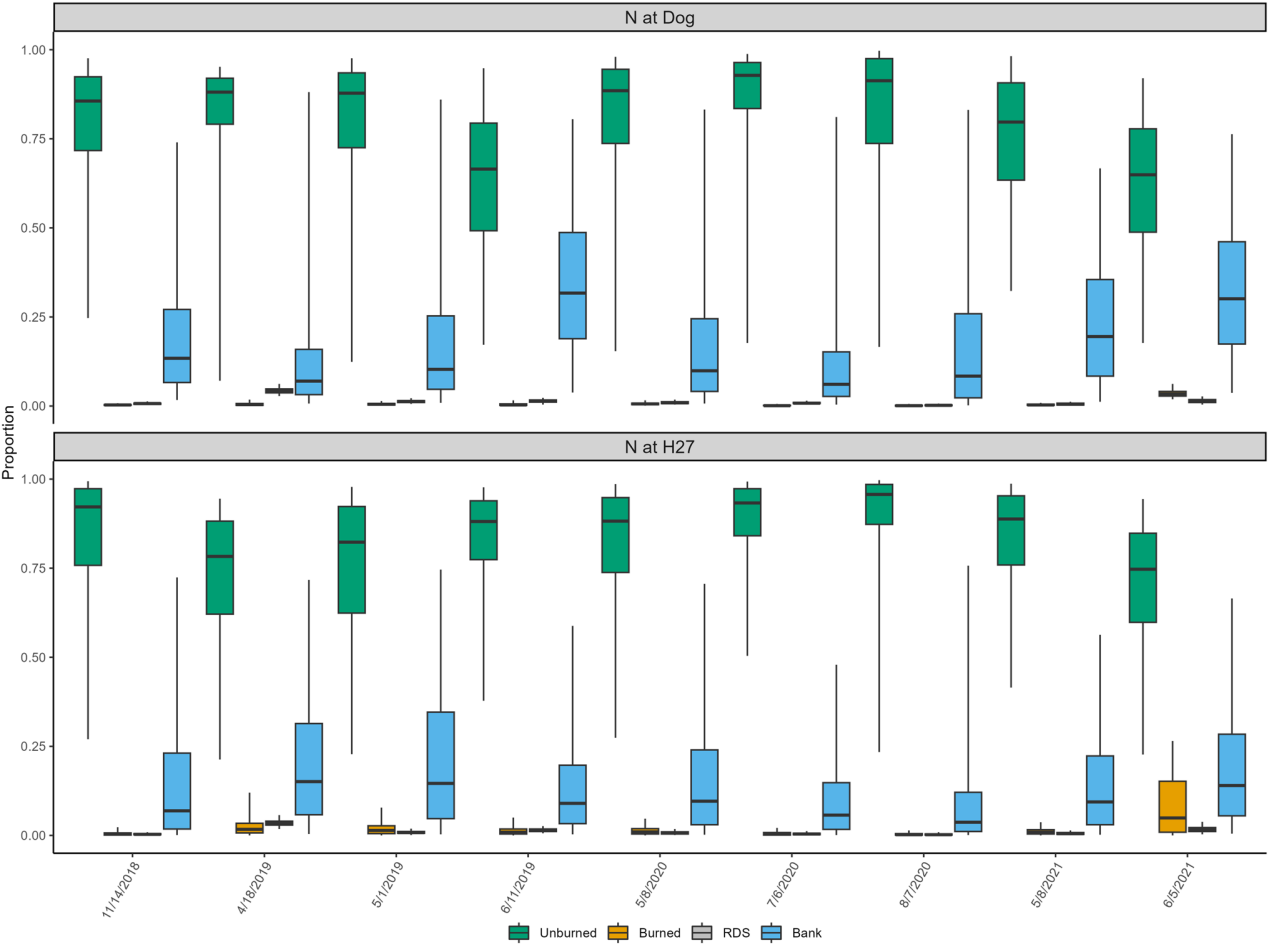
Proportional source contributions to sediment samples collected from the Nechako River mainstem sites using polycyclic aromatic hydrocarbon parameters for tracing

The same patterns of source apportionment for the tributary sites were consistent at the mainstem sites. As shown in Fig. 7, the results showed that at N@Dog, there was no contribution from burned sources or RDS, and the median values for bank contributions were only slightly more elevated. For the N@H27 site, the results were similar, as unburned soil contributed 74–96% with the remaining source material coming primarily from banks. It is of note that RDS contributed less than 1% according to the model output, compared to 9–48% contribution when color was used. Of note is that the only sampling date that showed a slightly elevated contribution from burned sources was on June 5, 2021, the last PAH sample collected.

### Evaluation of model output using virtual mixtures

Using color coefficients as tracers in the tributaries, the virtual mixtures were correct in their identification of the primary source of sediment using the median value in 80% of all cases, and this value increased to 87% under CI_95_. In the cases where the primary source was misidentified, it was second to the source most similar to it (e.g., burned topsoil and burned OM), and there were no cases where a primary source was misidentified as a source from the other broad (i.e., burned vs. unburned) source type. The overall mean absolute error was 14.5%. The primary trend in results from the virtual mixtures was that the model performed best when it was unmixing burned vs. unburned sources, and had issues when unmixing between similar sources (i.e., burned topsoil vs. burned OM). For example, a virtual mixture of 40% bank, 10% burned OM, 10% burned topsoil, and 40% unburned soil had an overall mean absolute error of 6% whereas a mixture that was 15% bank, 40% burned OM, 40% burned topsoil, and 5% unburned had an overall mean absolute error of 19%, with the largest error associated with burned OM being underrepresented. There was no trend in every mixture as to what source was overrepresented.

Results from the virtual mixtures using color coefficients in the mainstem produced similar results to the tributaries, identifying the primary source in 87% of mixtures using the median value. The prediction rate increased to 93% when including CI_95_. The primary trend was the tendency for the model output to underpredict the contribution from RDS samples, though it was able to consistently identify RDS as the primary source, just with elevated absolute errors. The overall mean absolute error for the mainstem sites was 12%, with bank samples the most correctly apportioned overall.

The analysis of virtual mixtures using PAHs was undertaken, though because they are a novel tracer, it is advised that laboratory mixtures or other methods, such as submersion experiments, should be used (Evrard et al. 2022). Due to the evidence presented in Sect. 3.2 that PAHs are likely not acting conservatively and have trouble discriminating between sources (Fig. 5), the results from the virtual mixtures are not used only to examine the ability of the model to correctly predict sources. As MixSIAR will always provide an unmixing solution, even if the input data are problematic, the virtual mixtures were used primarily to better understand the issues surrounding PAHs as tracers. The virtual mixtures from the tributaries exhibited a few trends that help explain the results using the field data. When burned topsoil was the primary source, the model showed mixed results in identifying it as such, and when it was correctly identified, the absolute error was large (33%). The clearest trend was that the model always overrepresented banks and unburned material and allocated similar values to them both, regardless of what the virtual mixture showed. For example, a virtual mixture of 60% unburned and 10% banks allocated 19.6% to unburned and 19.3% to banks.

When using the mainstem sources, there were a few trends that explain some of the results using the field data. First, the model was able to predict the primary source of sediment correctly almost exclusively when the primary source was banks or unburned material but was not accurate when it was RDS or burned material. In the cases where RDS was identified correctly as the primary source, the mean absolute error was 29%. The model was also biased towards always allocating to banks and unburned material in similar proportions, even when the virtual mixtures skewed towards one of these sources. For example, 39.6% was allocated to banks and 37.5% to unburned material when the virtual mixture was 10% bank and 60% unburned. This trend was always one-way, overrepresenting banks, and underrepresenting unburned material. Finally, the model consistently underrepresented both RDS and burned material, results that are similar to the findings from running the field samples, as described in Sect. 3.2.

## Discussion

### Tracer and modeling considerations

The following discussion on the primary sources of sediment following the Shovel Lake fire in the tributaries and the mainstem of the Nechako River (Sect. 4.2) is based on the results derived from using color properties as tracers. While one of the objectives of this paper was to assess the utility of both PAHs and color properties as tracers, there are several reasons why the results derived using PAHs are less reliable compared to those based on color properties. These reasons are further detailed in Sect. 4.4, but a primary reason is that unlike PAHs, color properties have been used widely in sediment fingerprinting studies and have been shown to act conservatively (Martínez-Carreras et al. 2010; Brosinsky et al. 2014; Barthod et al. 2015; Evrard et al. 2019a; Ramon et al. 2020; Amorim et al. 2021; Pulley and Collins 2021; García-Comendador et al. 2023). However, it is necessary to acknowledge that color has been rarely utilized in wildfire studies (García-Comendador et al. 2020), and therefore, Sect. 4.3 presents an assessment of its potential and some considerations for future research.

The use of MixSIAR has become a mainstay in the sediment fingerprinting literature, but it is important to note potential consequences that can come from its use without due diligence with respect to the number of sources and the number of samples per source. While much attention has been paid to choosing conservative and discriminatory tracers (e.g., Collins et al. 2020; Lizaga et al. 2020), less attention has been paid to the choice of sources themselves other than the acknowledgement that one should not use less than *n* − 1 tracers for *n* sources. In this study, the decision of whether to include subsurface soils as part of the unburned source category as well as whether to combine burned sources in the mainstem site was important due to the way it changed the total number of source samples, and how many samples were in each source category. Ultimately, the decision to combine unburned topsoil samples with burned subsoils was made because there was no significant difference in their values. The number of source samples was limited due to the high cost associated with PAH analysis, but in situations where there are numerous sediment samples and few source samples, there can be significant deviation allowed from the source and sample means, and if an unimportant source is included in the model with very few samples, it can be overrepresented in the mixture (Stock et al. 2018). Neither of these are criticisms of MixSIAR but instead are a reiteration that obtaining a large number of samples spread evenly across sources is important, and that careful consideration of source data is paramount.

As detailed in Sects. 3.1 and 3.2, MixSIAR output showed that, in the tributaries for burned OM in particular, the contribution was very low, often close to 1%. These values are not wholly inaccurate, but if taking the model uncertainties and errors into account, it is likely that these values reflect the fact that the burned OM is not a particularly important source and may not be acting conservatively. This is not to imply that color is non-conservative, but that burned organic matter itself may not be acting conservatively, due to the ability of organic material to degrade biologically, chemically, and physically (Certini 2005; Agbeshie et al. 2022).

The results from the virtual mixtures were useful in understanding how the modeling limitations, namely source discrimination, were manifest in the unmixing results, particularly for color. For the tributary sites, the color-based virtual mixtures showed that there were clear differences between the two burned sources and the two unburned sources. Additionally, the virtual mixtures showed that MixSIAR was successful in the identification of the main sources of sediment, a primary objective of this study. The same was true using the sources for the mainstem sites, though it is likely that the contribution from RDS has been underrepresented. The findings using PAHs in virtual mixtures reiterated the issues with their use as a tracer, as the model allocated to unburned material and banks almost equally, regardless of what the virtual mixture showed.

Ultimately, the results from the virtual mixtures are useful in reiterating the limitations of unmixing models when sources are similar, and in ensuring that results are not focused only on the median source apportionment values, but the entire range of values as shown in the proportional plots. Finally, while the use of virtual or laboratory mixtures can be beneficial in specific cases, there are other methods that may be used as alternate lines of evidence, which can include erosion models, submersion experiments, or prior knowledge of specific watersheds (Batista et al. 2022; Evrard et al. 2022).

### Source apportionment following the Shovel Lake wildfire

The results from the tributaries showed that the wildfire had a measurable impact on sediment sources, more so in Nine Mile and Tatsutnai Creeks than in Ormond Creek. The differences observed in Ormond Creek are likely for a number of reasons, including that there are three lakes within its watershed—Justine (area = 2.48 km^2^), Oona (area = 3.3 km^2^), and Ormond (area = 3.26 km^2^), the latter two of which are close to the Shovel Lake fire boundary. Thus, there would be a dampening effect on Ormond Creek from any impacts of the fire upstream of Oona and Ormond Lakes. This suggests that areas of sediment deposition and storage, such as lakes and wetlands, may buffer the downstream river system from the effects of wildfire. In addition, channel banks were a significant contributor of sediment in Ormond Creek, which may be a secondary effect of the fire, reflecting bank collapse due to the loss of root strength and vegetative cover (Eaton et al. 2010; Owens et al. 2012) and increased river discharge that normally follows wildfire (Certini 2005; Shakesby and Doerr 2006). The elevated contribution from banks in Ormond Creek is similar to results found by Gateuille et al. (2019) in nearby Greer Creek, which has similar characteristics (i.e., slope, cover) as Ormond. Additionally, construction of a natural gas pipeline was being undertaken in the Ormond Creek watershed in the summer/fall of 2021, which included excavation and large-scale movement of soils very near the creek for pipeline installation. This was downstream from the lakes but just upstream from the sampling location (Fig. 1) and could have played a role both as a point source of unburned material as well as a non-point source from banks as there were periods when the flow of Ormond Creek was altered (Coastal Gas Link 2021). However, this would only have affected the results of the samples collected in the summer and fall of 2021. Tatsutnai and Nine Mile Creeks are similar watersheds and their results are comparable, though Tatsutnai had a consistently higher contribution from burned topsoil, likely because it was more heavily burned than Nine Mile (76% and 47% burned, respectively). Nine Mile had a much higher percent of non-forested land (Table 1), which may also help explain both the smaller percentage of land burned and subsequently, the smaller contribution from burned areas. Tatsutnai and Nine Mile Creeks, with their many similarities and their lack of lakes that can mask the impact of fire in the immediate term, proved to be useful watersheds in gaining knowledge on the response of small creeks to significant disturbance from wildfire.

The Nechako mainstem site N@Dog was upstream of the outlets of Tatsutnai and Nine Mile Creeks but was downstream from the confluence of the Nautley River that drains Fraser Lake (Fig. 1), of which Ormond Creek is a large contributor. The low percentages of material derived from fire at this site were likely due to the dampening effect of Fraser Lake which reduces the connectivity between the upstream watershed and downstream channel network. The relatively high contribution from banks was not particularly surprising as there are large cutbanks at the confluence of the Nautley River, which is not far from the N@Dog site, while its remoteness and lack of paved roads explain the small contribution from RDS. However, as the virtual modeling showed, there was a small underrepresentation of RDS. Results from this study are in line with those from Gateuille et al. (2019), who found erosion of channel banks to be the greatest contributor to this site.

The N@H27 site was located directly beneath a road bridge that was used heavily by recreational and industrial traffic. Two RDS samples were collected directly from the road shoulder on this bridge, which had deposits on the bridge deck year-round. Additionally, it was common to observe RDS falling off the bridge and deposits could be seen on the land beneath the bridge, which provides another line of evidence that this contribution from RDS is expected at this site. Similar to the results for the N@Dog, it is likely that materials derived from the Shovel Lake wildfire were diluted by the time they made it to this site because of the significant inputs of water from Fraser Lake and numerous small creeks downstream from the impacted tributaries described in this paper. Finally, watershed response to wildfire, and erosion response in particular, depend on the post-fire precipitation regime as even high-severity fires can have a small geomorphic response when there are low intensity or very few hydrologic events immediately following the fire (Neary et al. 2005; Owens et al. 2013). In this study, there was 75.6 mm of rain and 8 mm of snow in the month prior to the first sample being collected (14 November 2018), the largest single rainfall event occurring on November 1–3 when 38.5 mm of rain fell (Environment and Climate Change Canada 2018). The dry preceding conditions and dry conditions after this rainfall event likely mean that there was not a significant amount of overland runoff that occurred, and that if there was an impact on the watershed, it likely came with the increase in discharge and thus, further bank erosion. After this event, there was little hydrologic activity, and daytime highs were below freezing, leading to frozen soils by mid-December and thus, a lack of major runoff events until the following spring.

### Assessment of color properties as sediment tracers

While it is not nearly as popular in the sediment source fingerprinting literature as geochemistry or fallout radionuclides, color properties have been gaining popularity as a tracer for several reasons. First, laboratory analysis is relatively simple and inexpensive and can be undertaken using a variety of equipment including office scanners. Second, the general consensus to date is that color properties behave conservatively and, at least thus far, can be used in a diverse set of landscapes (e.g., Martínez-Carreras et al. 2010; García-Comendador et al. 2020; Pulley and Collins 2021). Prior to this study, colour had only been used once in relation to wildfire (i.e., García-Comendador et al. 2020), but based on their results and those presented here, it appears to be a suitable tracer for determining sediment sources in wildfire-impacted watersheds. However, while there was no significant difference in color values between topsoil samples collected in 2018 and 2020 in this study, for 11 of 15 color coefficients, there was a significant difference between the 2018 and 2021 burned topsoil samples. It is important to note this is only for the burned topsoil samples, and that there was still discrimination between the burned and unburned (i.e., RDS, bank, unburned soil) sources. Thus, when using color in wildfire impacted areas, it would be useful to sample sources more than once if a study is going to be conducted over multiple years, which is feasible due to the low cost of the sample analysis (~ $15/sample). Ultimately, for source apportionment studies being undertaken by watershed managers or public interest groups, color is a highly useful, cost-effective option and should be considered in all landscape types. With the research here being a prescient example, those aforementioned characteristics also make color a very useful tool in determining the suitability of a new tracer.

### Assessment of the use of PAHs as sediment tracers

Using PAHs as tracers in sediment fingerprinting studies has not previously been undertaken, though there has been a growing interest in PAHs as a contaminant of terrestrial and aquatic systems after large wildfires (for a review, see Kieta et al. 2023a). The literature on tracing sediment after wildfire is also relatively sparse (Smith et al. 2013), and therefore, the significant fires in the NRB that impacted many of its tributaries presented a unique opportunity to determine the suitability of PAHs as tracers. Based on the success of using other organic tracers (e.g., fatty acids), the ubiquity of PAHs after wildfire, and the clear demarcation of burned vs. unburned areas, there was reason to believe they would prove to be a useful new tracer. The results presented above show that there is reason to believe that PAHs are not acting conservatively, even though some pass the range and Kruskal-Wallis tests.

While a single reason for their failure cannot be pinpointed with the data from this study, it is our belief that there are a few mechanisms occurring that undermine their conservatism. The first is their potential breakdown in the environment by photodegradation, which is most likely to impact two-ring and linear PAHs (Korfmacher et al. 1980). The second, and likely more important mechanism is microbial degradation. There has been no conclusive measure of the half-lives of PAHs, but some studies have determined that the half-life for two-ring PAHs is less than 10 days, for three-ring PAHs is less than 100 days, and for four and five–ring PAHs is more than 100 days (Herbes and Schwall 1978; McGinnis et al. 1988; Volkering and Breure 2003). Of the 16 EPA priority PAHs that were used in this study as possible tracers, 11 are high molecular weight (HMW) PAHs, meaning they are composed of four or more aromatic rings, and five are low molecular weight (LMW) PAHs, meaning they are composed of two or three rings. The differences between HMW and LMW compounds are numerous, most importantly in how they are produced and released to the environment and also in how they move through the environment (Connell 2005; De La Torre-Roche et al. 2009; Achten and Andersson 2015; Abdel-Shafy and Mansour 2016). The results of the range and Kruskal-Wallis tests identified eight PAHs suitable for use in MixSIAR, and of those eight, six were HMW and two were LMW. However, LMW PAHs are usually the dominant fraction produced during wildfire (Vila-Escalé et al. 2007; Kim et al. 2011; Rey‐Salgueiro et al. 2018; Chen et al. 2018). One clear signal of wildfire is the extremely elevated concentrations of Nap in burned soil samples (Vergnoux et al. 2011), but in this study it failed the range test and thus was not included in the mixing model. In general, LMW PAHs were found in higher concentrations than HMW compounds in sediments. Thus, the problem may be twofold: (1) rapid breakdown of LMW PAHs may render them non-conservative and therefore, unsuitable as a tracer, and (2) LMW PAHs are the dominant fraction post-wildfire but did not pass the tests for conservatism and were not used in MixSIAR.

The lower octanol–water partition coefficient (K_ow_) of LMW PAHs compared to that of HMW PAHs means that the latter is more likely to be found in the particulate form, while the former is mainly in the dissolved form, which may also be undermining the results. Additionally, chrysene, which can be an indicator of wildfire (Jiang et al. 1998), was excluded from the MixSIAR models due to it failing the range test. Other PAHs that are known to be produced primarily during wildfire rather than during the combustion of fossil fuels are cadalene, simonellite, retene, and 4,5 methylenephenanthrene (Wakeham et al. 1980; Yunker and Macdonald 1995; Baumard et al. 1998; Jiang et al. 1998; Yang 2000). However, these are not part of the list of 16 priority EPA PAHs and were not analyzed here due to additional costs (adding ~ $200 per sample). Thus, future studies may consider a reduced number of samples that includes a greater number of PAH compounds that can better elucidate wildfire sources.

Finally, while the wildfire did produce PAHs that were detectable in soils and sediments, the low concentrations of PAHs (Table 4) in the unburned areas was unsurprising due to the remote nature of the NRB, its lack of large industrial sources, and the low number of inhabitants, and thus, lack of a major urban center. However, these low concentrations likely are another part of the reason that PAHs did not work as a tracer as PAHs that did make it to the Nechako mainstem were significantly diluted (and potentially already breaking down), and thus, not feasible to trace back to the burned areas. The low PAH concentrations are important for two reasons: (i) they are a sign that in remote areas even large wildfires may not produce levels of PAHs that are toxic to aquatic organisms, and (ii) they are not produced in high enough concentrations to be used as a tracer given present analytical equipment (i.e., LODs). However, while the low concentrations may have hindered their use as a tracer, they were useful as a determinant of environmental pollution post-wildfire. Concentrations in the tributaries and the mainstem Nechako River varied, and as determined in Kieta et al. (2023b), the sources of PAHs also varied. That study found that vehicular emissions were a greater contributor to PAHs than wildfire in the mainstem, whereas the tributaries showed a greater wildfire signal. In addition to PAH source apportionment, the authors found that concentrations of PAHs in soils had decreased from 2018 to 2021 and were below soil quality guidelines but were not yet back to concentrations found in unburned soils (Kieta et al. 2023b ).

**Table 4:** Mean and standard deviation for 16 EPA priority PAHs in source and sediment samples

Analyte	Sediment (ng g^−1^)	Source (ng g^−1^)
Acenaphthylene	0.23 (0.39)	12.2 (32.0)
Acenaphthene*	0.27 (0.32)	5.2 (12.1)
Anthracene*	0.33 (0.39)	9.7 (22.6)
Benz[a]anthracene*	0.65 (0.59)	5.3 (11.0)
Benzo[a]pyrene	0.49 (0.63)	4.4 (11.6)
Benzo[b]fluoranthene	1.26 (1.0)	4.8 (11.5)
Benzo[ghi]perylene*	1.03 (0.93)	4.7 (10.6)
Benzo[j,k]fluoranthene*	0.47 (0.53)	3.9 (10.2)
Chrysene	2.48 (1.73)	12.6 (20.6)
Dibenz[a,h]anthracene	0.34 (0.21)	0.82 (1.8)
Fluoranthene*	2.42 (2.27)	20.6 (41.0)
Fluorene	0.84 (0.66)	19.1 (47.7)
Indeno[1,2,3-cd]pyrene*	0.59 (0.56)	3.2 (8.8)
Naphthalene	5.01 (4.8)	617.4 (1484.0)
Phenanthrene	8.35 (6.28)	96.3 (205.3)
Pyrene*	2.25 (2.06)	16.0 (30.6)

Source samples include road deposited sediment, banks, and topsoil from the burned and unburned sites from 2018 only

*Denotes PAHs used in the MixSIAR model

It is difficult to know exactly how PAHs might be changing as they move through a watershed, particularly when discussing their degradation with respect to microbial communities in both the terrestrial and aquatic environments. Gateuille et al. (2014) aimed to quantify the decontamination times of PAHs in a catchment outside of Paris, France, and found that degradation and volatilization were the main processes of removal of PAHs and that, in their estimation, degradation rates were much longer than those reported elsewhere. However, this study was undertaken in an area with significantly more industrial and urban input than the research presented here. Furthermore, wildfires complicate degradation rates and transfer from the terrestrial to the aquatic environment because of the significant impact fire has on soils, particularly on soil organic matter. Numerous studies have shown that severe wildfire has the short-term impact of reducing microbial biomass (Hernández et al. 1997; Hebel et al. 2009; Whitman et al. 2019) and that microbial populations may (Prieto-Fernández et al. 1998) or may not (Ferrenberg et al. 2013; Dove et al. 2020) rebound to their pre-fire levels. Based on the physical and chemical properties of PAHs (i.e., PAH structure and ring number, octanol–water partition coefficient (K_ow_), organic carbon–water partition coefficient (K_oc_)) and the impacted soils (i.e., carbon content, light vs. dark ash, etc.), there is a range of mechanisms by which microorganisms, bacteria, and fungi can degrade PAHs. While each of these specific pathways is not fully understood (see reviews by Samanta et al. 2002; Haritash and Kaushik 2009; Sakshi et al. 2020), the importance of microbial, fungal, and algal communities in the degradation of PAHs in the terrestrial environment has been well documented (see reviews by Cerniglia 1992; Haritash and Kaushik 2009; Ghosal et al. 2016). These properties, both of the microorganisms and the soils they inhabit, are vital in understanding the degradation of PAHs and how these properties of PAHs potentially translate to their conservative or non-conservative behavior as a tracer in fingerprinting studies. It is thus an area requiring significantly more research and examination.

Issues with respect to the conservatism of tracers are not a problem unique to PAHs, as this has also been a wide discussion with the use of CSSIs. Much has been written about the timing of sampling when using CSSIs as tracers because of the impact it can ultimately have on apportionment results (Reiffarth et al. 2016; Collins et al. 2019). For example, Reiffarth et al. (2019) demonstrated that the values of various CSSIs properties changed during seasons and over years reflecting variations in climate, crop/vegetation types, and land management practices (i.e., they were not conservative over time). This is an issue that arose with the use of PAHs as a tracer in this study, as there was a significant difference between concentrations in source samples collected in 2018 and 2021. While some of this may be attributed to their removal via erosion, it also reflects in situ degradation. This creates issues for tracing in that source samples from 2018 are not suitable for tracing sediment samples collected in 2020 or 2021. Thus, another consideration in the use of PAHs as a tracer is to plan source and sediment collection carefully with the acknowledgement that even sampling annually for sources may not be sufficient. 

## Conclusions

Under future climate scenarios, wildfires are predicted to occur over a longer season and to burn larger areas. However, due to the heterogeneity of soils, precipitation regimes, and topography of burned areas, predicting the contributions of sediment to waterways is not always straightforward. This study aimed to determine the sediment contributions from burned sources to three heavily burned creeks and the mainstem Nechako River after a large wildfire. While the results showed that there was little evidence of burned material in the mainstem, there was a significant contribution in the tributaries. The reason for the lack of wildfire signal in the mainstem sites was likely a combination of the disconnectivity caused by lakes and the dilution by non-wildfire sources such as channel banks and RDS in the case of the downstream site on the Nechako mainstem. In addition, the NRB is not as steep as some mountainous regions where fires occur, and it did not receive the significant amounts of rainfall that are often required to produce runoff immediately post-fire.

Apportioning sediment sources using a simple, inexpensive tracer such as color is a useful way to determine the impact of wildfires. Although PAHs did not work as a tracer in this study, they should not be disregarded completely as further research about the processes that cause their degradation in the environment could prove to be useful in elucidating circumstances where they could be used for fingerprinting. Additionally, due to their potential negative impacts on aquatic organisms, they should continue to be studied in relation to wildfires, particularly in areas where there are already elevated concentrations of PAHs due to urban and industrial activity. Finally, this study produced a range of questions with respect to the use of PAHs as tracers, and thus, the intention in reporting these results is to foster debate as to why PAHs may not have worked, and what can be done methodologically to facilitate their use in the future. .

### Supplementary Information

Below is the link to the electronic supplementary material.

Supplementary file1 (DOCX 14 KB)

## Data Availability

The data generated during the current study are available from the corresponding author on reasonable request.
