# Supplementary material for: Determination of sediment sources following a major wildfire and evaluation of the use of color properties and polycyclic aromatic hydrocarbons (PAHs) as tracers
Source: J Soils Sediments. 2023 Jul 4;23(12):4187–207. doi: 10.1007/s11368-023-03565-0 (PMC10684618; doi:10.1007/s11368-023-03565-0)
Supplement: Supplementary file 1 — Supplementary file1 (DOCX 14 KB) [file 11368_2023_3565_MOESM1_ESM.docx]

Supplementary information

Determination of sediment sources following a major wildfire and evaluation of the use of colour properties and polycyclic aromatic hydrocarbons (PAHs) as tracers

Kieta, K.A.^1*^, Owens, P.N.^2^, and Petticrew, E.L.^2^

Table S1

Table S1 Synthesis table outlining the type of source and sediment samples collected, number of samples analysed for each tracer type, and the samples that were included in MixSIAR

|  | Sample Type | Year(s) sampled | PAH analysis? | Colour analysis? | Included in MixSIAR? | Notes: |
| --- | --- | --- | --- | --- | --- | --- |
| Source | Burned topsoil | 2018, 2020, 2021 | Y; n=15 | Y; n=5 | 2018 n=5 | 2020 and 2021 samples used for contaminant analysis (Kieta et al., 2023) |
| Source | Burned OM | 2018 | Y; n=5 | Y; n=5 | 2018 n=5 | Combined with burned topsoil for mainstem sites |
| Source | Unburned soil | 2018 | Y; n=5 | Y; n=5 | 2018 n=5 |  |
| Source | Bank | 2018 | Y; n=5 | Y; n=10 | 2018 n=5 |  |
| Source | RDS | 2018 | Y; n=5 | Y; n=5 | 2018 n=5 |  |
| Sediment | Tributaries | 2018-2021 | n= 27 | n=73 | 2018-2021 |  |
| Sediment | Mainstem | 2018-2021 | n=18 | n=40 | 2018-2021 |  |
